# Multivariate lesion symptom mapping for predicting trajectories of recovery from aphasia

**DOI:** 10.1093/braincomms/fcae024

**Published:** 2024-02-01

**Authors:** Deborah F Levy, Jillian L Entrup, Sarah M Schneck, Caitlin F Onuscheck, Maysaa Rahman, Anna Kasdan, Marianne Casilio, Emma Willey, L Taylor Davis, Michael de Riesthal, Howard S Kirshner, Stephen M Wilson

**Affiliations:** Department of Hearing and Speech Sciences, Vanderbilt University Medical Center, Nashville, TN 37232, USA; Department of Hearing and Speech Sciences, Vanderbilt University Medical Center, Nashville, TN 37232, USA; Department of Hearing and Speech Sciences, Vanderbilt University Medical Center, Nashville, TN 37232, USA; Department of Hearing and Speech Sciences, Vanderbilt University Medical Center, Nashville, TN 37232, USA; Department of Hearing and Speech Sciences, Vanderbilt University Medical Center, Nashville, TN 37232, USA; Department of Hearing and Speech Sciences, Vanderbilt University Medical Center, Nashville, TN 37232, USA; Department of Hearing and Speech Sciences, Vanderbilt University Medical Center, Nashville, TN 37232, USA; Department of Hearing and Speech Sciences, Vanderbilt University Medical Center, Nashville, TN 37232, USA; Department of Radiology and Radiological Sciences, Vanderbilt University Medical Center, Nashville, TN 37232, USA; Department of Hearing and Speech Sciences, Vanderbilt University Medical Center, Nashville, TN 37232, USA; Vanderbilt Stroke and Cerebrovascular Center, Vanderbilt University Medical Center, Nashville, TN 37232, USA; Department of Neurology, Vanderbilt University Medical Center, Nashville, TN 37232, USA; Department of Hearing and Speech Sciences, Vanderbilt University Medical Center, Nashville, TN 37232, USA; School of Health and Rehabilitation Sciences, University of Queensland, Brisbane, QLD 4072, Australia

**Keywords:** aphasia, stroke

## Abstract

Individuals with post-stroke aphasia tend to recover their language to some extent; however, it remains challenging to reliably predict the nature and extent of recovery that will occur in the long term. The aim of this study was to quantitatively predict language outcomes in the first year of recovery from aphasia across multiple domains of language and at multiple timepoints post-stroke. We recruited 217 patients with aphasia following acute left hemisphere ischaemic or haemorrhagic stroke and evaluated their speech and language function using the Quick Aphasia Battery acutely and then acquired longitudinal follow-up data at up to three timepoints post-stroke: 1 month (*n* = 102), 3 months (*n* = 98) and 1 year (*n* = 74). We used support vector regression to predict language outcomes at each timepoint using acute clinical imaging data, demographic variables and initial aphasia severity as input. We found that ∼60% of the variance in long-term (1 year) aphasia severity could be predicted using these models, with detailed information about lesion location importantly contributing to these predictions. Predictions at the 1- and 3-month timepoints were somewhat less accurate based on lesion location alone, but reached comparable accuracy to predictions at the 1-year timepoint when initial aphasia severity was included in the models. Specific subdomains of language besides overall severity were predicted with varying but often similar degrees of accuracy. Our findings demonstrate the feasibility of using support vector regression models with leave-one-out cross-validation to make personalized predictions about long-term recovery from aphasia and provide a valuable neuroanatomical baseline upon which to build future models incorporating information beyond neuroanatomical and demographic predictors.

## Teaser

Levy *et al.* use machine learning to predict language outcomes across the first year of recovery from aphasia post-stroke, explaining nearly two-thirds of the variance in language outcomes and additionally demonstrating the importance of lesion location in making good predictions. See aphasia-friendly version of findings within!

## Graphical Abstract

**Graphical Abstract:**
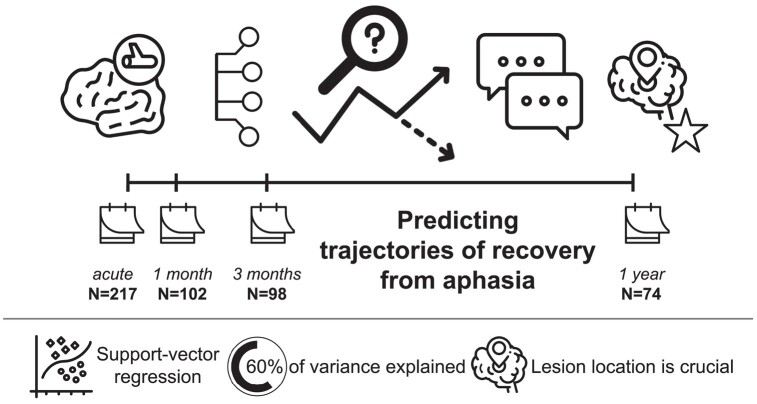

## Introduction

For an aphasia-friendly version of this paper, please see Supplementary Fig. 1.

Aphasia, an acquired disorder of language, is a common and debilitating consequence of stroke. Most individuals with post-stroke aphasia experience some degree of recovery of their language function, with the majority of gains occurring within the first year^1-4^; however, there is marked variability in the extent to which this recovery occurs.^1,4-7^ Previous work investigating factors contributing to aphasia recovery has demonstrated that lesion location and extent—particularly in left hemisphere perisylvian regions—are the clearest predictors of long-term language outcomes,^4,8-18^ with demographic information providing minimal predictive utility.^19-22^ Initial language presentation has also been reported as a powerful predictor of long-term outcome,^2,3,5,12,23^ though this measure is primarily a function of lesion location and extent.

The aim of the present study is to quantitatively predict language outcomes longitudinally in the first year of recovery from aphasia, across multiple domains of language and at multiple timepoints post-stroke. The ability to make such predictions is important for clinical reasons, such as providing data-driven expectations to patients and their loved ones and increasing clinicians’ ability to anticipate treatment needs in the context of clinical care. It is also important for neuroscientific reasons, as patterns of predictive utility of a model across language functions can provide insight into the extent to which distinct language subdomains can be mapped onto distinct neural substrates. Finally, such a model could provide a baseline upon which to assess the relative influence of other factors such as functional reorganization on long-term language outcomes.

The majority of studies that have examined relationships between patterns of brain damage and language outcomes have been carried out in chronic cohorts.^24-29^ While these prior studies have aided greatly in our understanding of lesion–outcome relationships at a broad level, many have been limited by coarse metrics of aphasia,^14,25,26^ relatively small cohorts^30^ or modest predictive utility.^24,27-29^ Though other studies have investigated recovery from aphasia longitudinally, most have not included image-based metrics among their predictors.^2,3,5,6,8,23^ Thus, no existing studies have aimed to account for the multidimensional and highly variable nature of aphasia recovery in a simultaneously longitudinal, lesion-informed, comprehensive and reliable manner.

Here, we use support vector regression (SVR) to predict scores on a multidimensional language battery at multiple timepoints post-stroke using demographic, language, lesion extent and lesion location–based predictors as input.

## Materials and methods

### Participants

A total of 217 individuals with aphasia were included in this study. All patients presenting at the Vanderbilt Stroke and Cerebrovascular Center at Vanderbilt University Medical Center were considered for inclusion. For our broader aphasia recovery project of which this study is a part,^4^ our inclusion criteria were (i) acute ischaemic or haemorrhagic stroke predominantly confined to left hemisphere supratentorial regions, or right hemisphere stroke with aphasia clearly indicating right hemisphere language dominance; (ii) age 18–90 years; and (iii) infarct at least 1 cm^3^ with the following exceptions: (i) thalamic infarcts were included regardless of extent, and (ii) starting after ∼21 months of data collection, basal ganglia and/or subcortical white matter infarcts were included only if they exceeded ∼6 cm. Our exclusion criteria were (i) unconscious with grave prognosis; (ii) not fluent in English premorbidly; (iii) prior symptomatic stroke significantly impacting language regions or homotopic regions, neurodegenerative disease or any other neurological condition impacting language or cognition; (iv) major psychiatric disorder; and (v) substance abuse serious enough to interfere with study participation. One thousand and fifty-five patients met the first inclusion criterion and were evaluated for inclusion, and ultimately, 354 met all criteria and consented to participate.^4^ For the present analysis, we focused only on patients who presented with aphasia acutely (*n* = 218), but we excluded one patient who had only mild aphasia despite an extensive left middle cerebral artery lesion, representing clear evidence for right hemisphere language lateralization, yielding our final sample of 217 individuals.

### Speech and language evaluations

**Figure 1:**
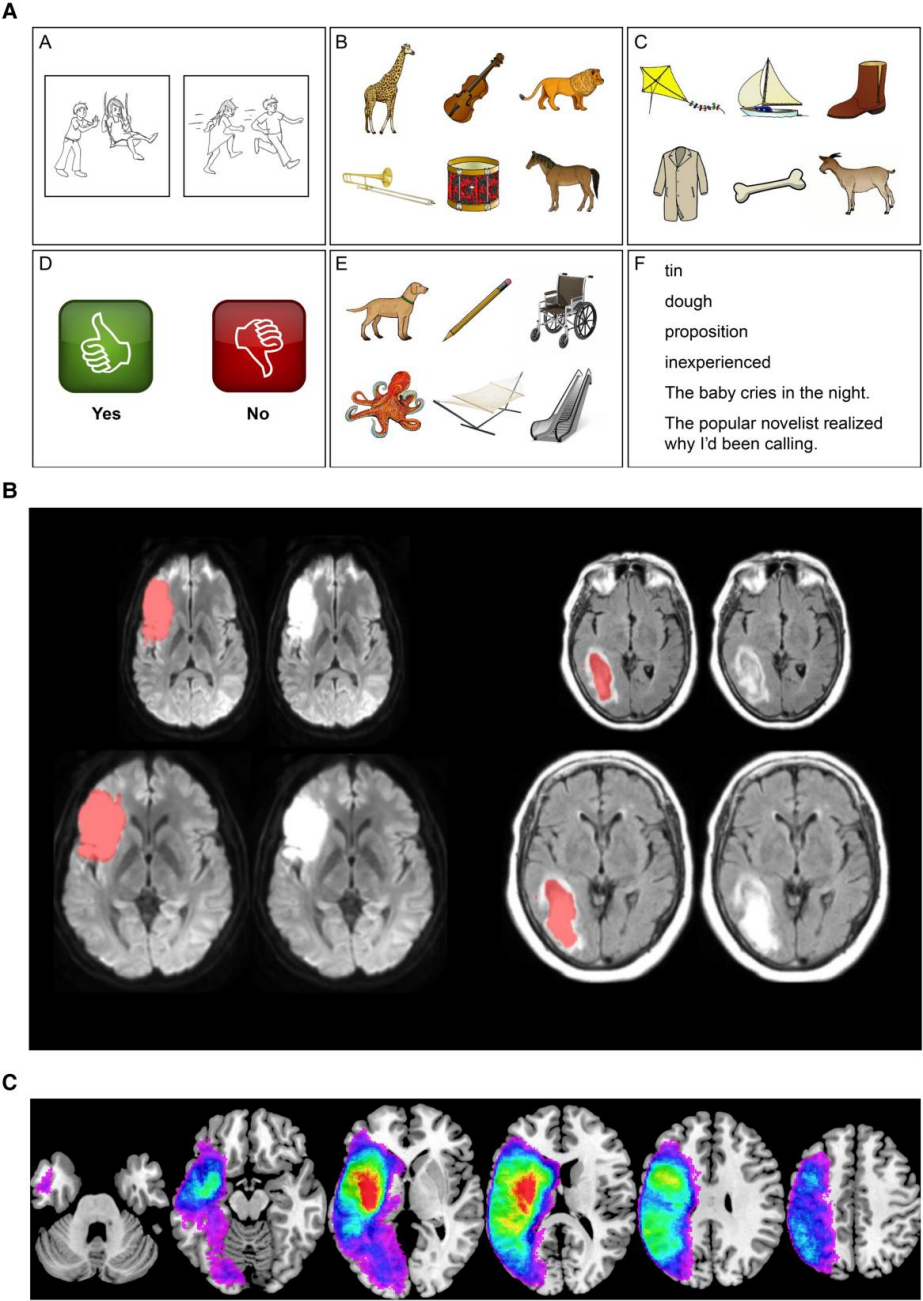
**Overview of methods.** (**A**) Example slides from the QAB^31^ (used with permission from copyright holder). (**B**) Examples of manual delineation (*top*) and normalization (*bottom*) on different imaging types; left shows diffusion weighted imaging as used for ischaemic strokes and right shows fluid-attenuated inversion recovery imaging as used for haemorrhagic strokes. (**C**) Overlay of lesions included in full data set.

Speech and language evaluation was completed at each timepoint using the Quick Aphasia Battery (QAB^31^; Fig. 1A). The QAB is a valid, reliable and time-efficient aphasia assessment consisting of eight subtests, from which a QAB overall score is derived, as well as seven subscores reflecting speech and language domains: single-word comprehension, sentence comprehension, word finding, grammatical construction, speech motor programming (i.e. absence of apraxia of speech), repetition and reading. We also examined speech motor execution, i.e. absence of dysarthria, which is scored as part of the QAB but does not contribute to the overall score. Scores vary on a scale from 0 (complete impairment) to 10 (no impairment/normal performance). Patients who were untestable at early timepoints but presumed (later confirmed) to be aphasic were assigned a QAB overall score of 0 (maximally impaired), while their subscores were treated as missing. Subscores were occasionally missing at other timepoints for various idiosyncratic reasons, e.g. limited baseline reading ability preventing assessment of reading difficulties due to stroke. These scores were treated as missing, and modified procedures were used to calculate QAB overall scores where necessary.^4^ All language evaluations were administered by certified speech-language pathologists (authors J.L.E., S.M.S. or C.F.O.).

QAB evaluations were sought from all eligible patients within the first 5 days after stroke. For those patients who presented with aphasia on initial evaluation or were untestable acutely and presumed likely to have aphasia, follow-up evaluations were sought at 1 month, 3 months and 1 year post-stroke. Note that, while the QAB defines the quantitative cut-off for aphasia as a QAB overall score of 8.9, diagnoses of aphasia were made using clinical impression as the gold standard. Of the 217 individuals with aphasia included in the study, 199 were formally tested using the QAB while 18 were untestable acutely but were found to be aphasic on follow-up (mean overall score at 1 month = 4.93 ± 2.33, range 0–8.05). The majority of these patients had extensive left hemisphere lesions (mean lesion size = 146.75 cm^3^, SD = 107.22 cm^3^, range = 6.32–376.56 cm^3^).

Among individuals who were testable acutely, there was no difference in initial severity between patients for whom follow-up data were obtained (mean overall score = 5.57 ± 2.69) versus not obtained [mean overall score = 6.04 ± 2.68, *t*(197) = 1.25, *P* = 0.21]. Demographic information at each timepoint is available in Table 1. There was no difference in the distribution of initial scores among the followed-up patients at any timepoint, suggesting no sampling bias towards patients who were initially less impaired in the longitudinal data (Supplementary Fig. 2).

**Table 1:** Demographic and clinical information reflecting patients included in the models at each timepoint

	Acute (*N* = 217)	One month (*N* = 102)	Three months (*N* = 98)	Twelve months (*N* = 74)
Age	62.5 ± 13.6 (21–90) years	61.9 ± 13.7 (21–90) years	62.2 ± 13.6 (23–84) years	61.9 ± 13.8 (23–90) years
Sex	117 M; 100 F	59 M; 43 F	57 M; 41 F	40 M; 34 F
Handedness	193 R; 19 L; 5 A	90 R; 10 L; 2 A	86 R; 9 L; 3 A	64 R; 7 L; 3 A
Education	12.9 ± 3.2 (0–20) years	13.2 ± 2.7 (3–20) years	13.2 ± 2.8 (3–20) years	13.6 ± 2.9 (3–20) years
Stroke type	174 I; 43 H	82 I; 20 H	78 I; 20 H	57 I; 17 H
Lesion extent	53.6 ± 60.4 (0.6–376.4) cm^3^	65.9 ± 71.1 (0.73–376.4) cm^3^	61.7 ± 71.2 (0.73–376.4) cm^3^	65.3 ± 72.9 (0.73–307.0) cm^3^
Acute overall severity	5.8 ± 2.7 (0–9.8)	5.5 ± 2.8 (0–9.8)	5.7 ± 2.7 (0–9.8)	5.4 ± 2.6 (0–9.8)

M, male; F, female; R, right; L, left; A, ambidextrous; I, ischaemic; H, haemorrhagic.

Audio and video were recorded for all sessions, which were then transcribed, scored and reviewed in consensus meetings attended by four to six authors.

### Neuroimaging

As part of their clinical care, all patients who come through Vanderbilt University Medical Center suspected for stroke undergo a brain MRI and/or head CT to identify the presence, location and extent of neural damage. Lesions were delineated manually on these images by trained personnel (authors D.F.L. and M.R.; Fig. 1B). Coregistration and normalization of lesions were carried out as described in Wilson *et al*.^4^ prior to smoothing with an 8 mm full width at half maximum Gaussian kernel. An overlay of the resulting lesion masks for the full data set is displayed in Fig. 1C.

**Figure 2:**
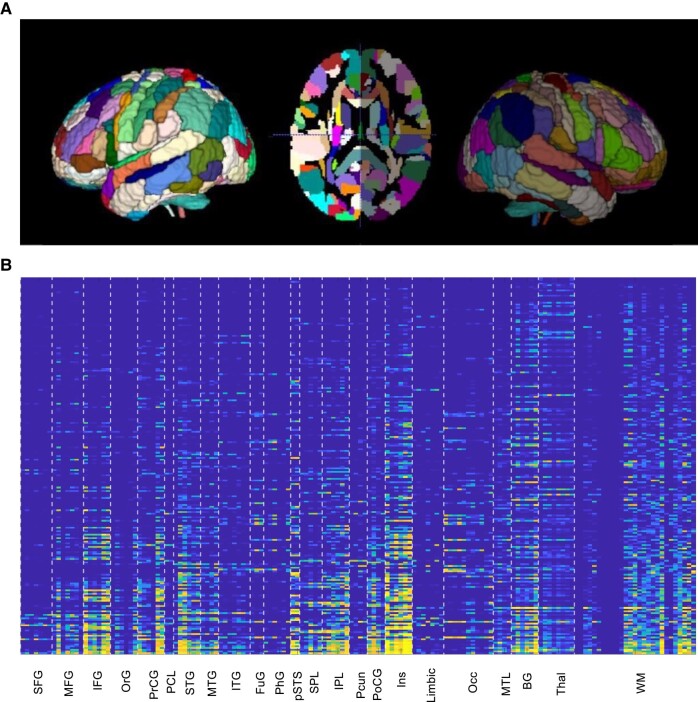
**Generation of LLVs.** (**A**) Combined grey and white matter atlas used for LLV generation. Note that only left hemisphere ROIs were used. (**B**) LLVs for the full data set of 217 patients (rows) by 150 ROIs (columns), rows/patients sorted in ascending order of lesion size. SFG, superior frontal gyrus; MFG, middle frontal gyrus; IFG, inferior frontal gyrus; OrG, orbital gyrus; PrCG, precentral gyrus; PCL, paracentral lobule; STG, superior temporal gyrus; MTG, middle temporal gyrus; ITG, inferior temporal gyrus; FuG, fusiform gyrus; PhG, parahippocampal gyrus; pSTS, posterior superior temporal sulcus; SPL, superior parietal lobule; IPL, inferior parietal lobule; Pcun, precuneus; PoCG, postcentral gyrus; Ins, insula; Limbic, limbic structures; Occ, occipital; MTL, medial temporal lobe; BG, basal ganglia; Thal, thalamus; WM, white matter.

The resulting lesion masks were transformed into vector space representations, henceforth referred to as lesion load vectors (LLVs), via calculation of the overlap of each patient’s lesion mask with 150 spatial regions of interest (ROIs) in the left hemisphere of a custom combined grey matter and white matter atlas (based on Mori *et al*.^33^ and Fan *et al*.^34^; Fig. 2A). This atlas was designed to afford sufficient granularity across broad swaths of language cortex that are known to be heterogeneous in nature,^35^ in particular the ability to distinguish between the superior temporal sulcus and the adjacent superior and middle temporal gyri. The resulting atlas consisted of 123 left hemisphere grey matter ROIs, 21 left hemisphere white matter ROIs and the left hemisphere portions of six commissural tracts. Each patient’s LLV consisted of 150 values between 0 and 1 representing the proportion of each ROI that was lesioned (Fig. 2B).

### Statistical analysis

#### Model fitting

SVR with a linear kernel was chosen to model relationships between predictors and language scores due to its ability to handle high-dimensional input data, lack of sensitivity to outliers and resistance to overfitting.^36,37^ We sought to follow best practices in multivariate lesion symptom mapping (e.g. full independence of training/testing data and use of appropriate metrics of prediction accuracy; see Scheinost *et al*.^38^ for details).

Two main sets of models were constructed to predict QAB overall and each of the eight domain-specific subscores at each timepoint.

The first set of models will be referred to as LLV models. These models attempted to predict speech/language measures based on lesion location, as encoded in the 1 × 150 LLVs. Also included in the models were lesion extent, age, sex, handedness, years of education and stroke type (ischaemic/haemorrhagic). All of these additional variables were min–max scaled.

The second set of models will be referred to as LLV + initial presentation (LLV + IP) models. These models contained the same explanatory variables just described but also included patients’ overall scores at the initial timepoint. Because initial scores were included as inputs, these models were only constructed for the 1-month, 3-month and 1-year timepoints. This set of models reflects potential clinical applications, in which lesion location and IP are known, and the goal is to predict subsequent trajectories.

Two sets of reduced models were also generated for each of the LLV and LLV + IP models: the first excluding LLVs (so that prediction was based only on lesion extent, demographic and stroke type variables, plus IP in the case of LLV + IP) and the second also excluding lesion extent (so that prediction was based solely on demographic and stroke type variables, plus IP in the case of LLV + IP).

All models were fit using the ‘fitrsvm’ function in Matlab2022b using the default parameters for linear SVR (box constraint = 1, epsilon = interquartile range of response variable/13.49 and gamma = 1). Following model fitting, predictions were capped to the range of possible scores (0–10).

#### Assessment of predictive accuracy

Model generalizability was evaluated using a leave-one-out cross-validation procedure, in which each patient was held out in turn to have their score predicted from a model based on data from the remaining patients.

Model performance was evaluated using prediction *r*^2^ as defined in Alexander *et al*.,^39^ corresponding to the ratio of the difference between each observed value and its predicted value compared to the difference between each observed value and the mean (that is, how much better the model performs than simply guessing the mean response value). Note that prediction *r*^2^ is more conservative than the oft-reported squared correlation coefficient; note also that prediction *r*^2^ can be negative in cases where the model performs worse than predicting the mean, which may occur in threshold-based model-fitting procedures such as epsilon-insensitive SVR when predictors are not actually informative.

Prediction *r*^2^ is a particularly conservative metric in the context of ceiling effects, as it is penalized in a manner that increases with decreasing variance in the observed data^39^; therefore, predictive accuracy will be assessed as worse when the true scores to be predicted fall within a narrow range. We report root mean squared error in Supplementary Tables 1 and 2 as a complementary metric to reflect raw prediction accuracies unaffected by underlying variance.

#### Topographic mapping using feature weights

In order to investigate the potential neural bases of long-term greater aphasia severity, feature weights (i.e. model regression coefficients) in which higher values of the predictor were associated with lower QAB overall scores were extracted from the LLV model at the 1-year timepoint with a threshold of 1 SD from the mean feature weight. Note that there are currently no agreed-upon guidelines for assessing the statistical significance of SVR-based beta weights,^40^ and thus, these features serve only as a preliminary means of understanding some of the neural regions that may play the biggest role in the prediction of aphasia outcomes.

## Results

For a descriptive account of trajectories of recovery across the data set at large, see Wilson *et al*.^4^ (Note that slight discrepancies in reported numbers are due to exclusion of one patient with clear right hemisphere language lateralization in the current paper.)

### LLV models

These models included information about lesion location and extent, as well as age, sex, handedness, education and stroke type, but no information about IP.

**Figure 3:**
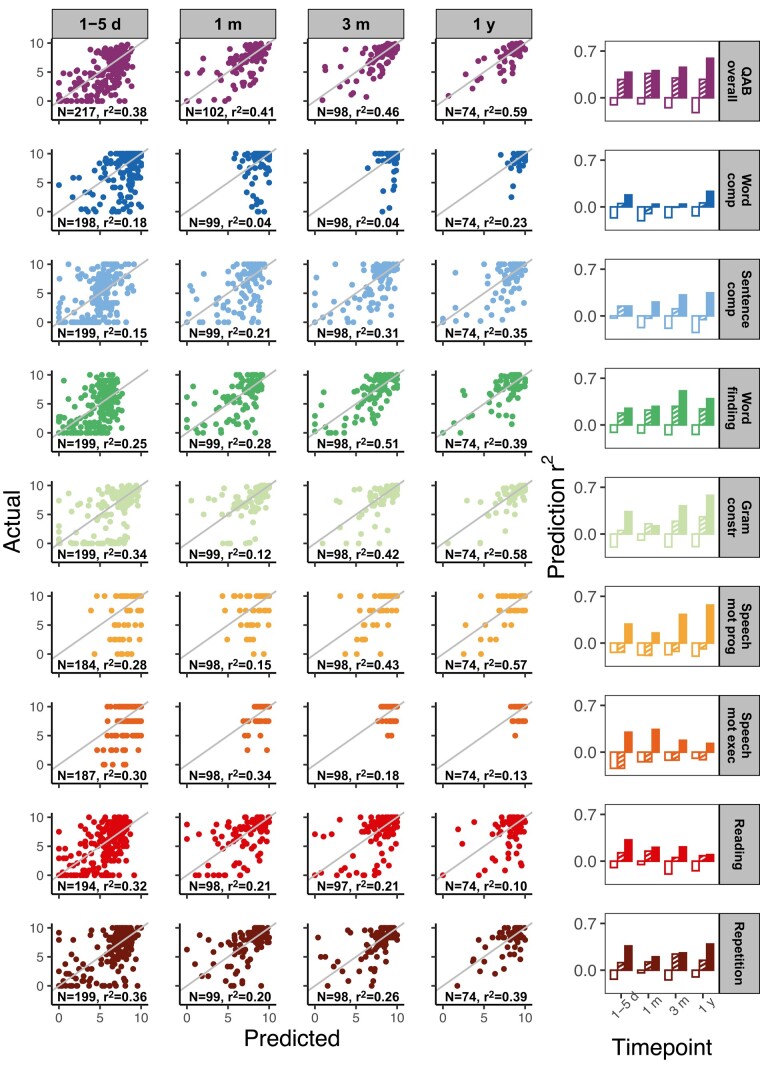
**Model performance for the lesion load models.** (Left) Scatter plots comparing actual (*y*-axis) and predicted (*x*-axis) scores on the QAB overall as well as eight subscores (rows) in models using lesion load, lesion size and demographic information as predictors. The four columns show the acute (1–5 days post) timepoint, 1-month timepoint, 3-month timepoint and 12-month timepoint. Sample size and prediction *r*^2^ are displayed for each model. Grey identity lines are plotted for reference to show how perfect prediction accuracy would appear. (Right) Bar plots showing prediction *r*^2^ across all timepoints for QAB overall and the eight subscores (rows). Unfilled bars correspond to models using demographic-only predictors, shaded/striped bars correspond to models using demographic and lesion size predictors and solid bars correspond to models using demographic, lesion size and lesion load/location predictors. Sample sizes for each group of bars within a plot are equal and match those listed on the scatter plot for the corresponding subscore and timepoint. QAB overall, Quick Aphasia Battery overall score; Word comp, single-word comprehension; Sentence comp, sentence comprehension; Gram constr, grammatical construction; Speech mot prog, speech motor programming; Speech mot exec, speech motor execution.

QAB overall was predicted with *r*^2^ = 0.38 at the acute timepoint, *r*^2^ = 0.41 at the 1-month timepoint, *r*^2^ = 0.46 at the 3-month timepoint and *r*^2^ = 0.59 at the 1-year timepoint (Fig. 3). The LLVs were critical to this good performance, since the full models outperformed models including lesion extent but not location (acute: reduced *r*^2^ = 0.27; 1 month: reduced *r*^2^ = 0.36; 3 months: reduced *r*^2^ = 0.30; 1 year: reduced *r*^2^ = 0.28).

Reduced models including only demographic and stroke type information had little to no predictive power, as expected.

Predictive power varied for the nine QAB subscores (Fig. 3, Supplementary Table 1). Word finding and grammatical construction were predicted particularly well across all timepoints, while single-word comprehension, speech motor execution and reading proved more difficult to predict.

LLVs improved performance in 34 out of 36 (timepoints by subscores) cases, indicating that specific information about the lesion site is critical to optimize prediction.

### LLV + IP models

These models included information about IP (as measured by QAB overall at the acute timepoint) along with lesion location and extent, age, sex, handedness, education and stroke type.

**Figure 4:**
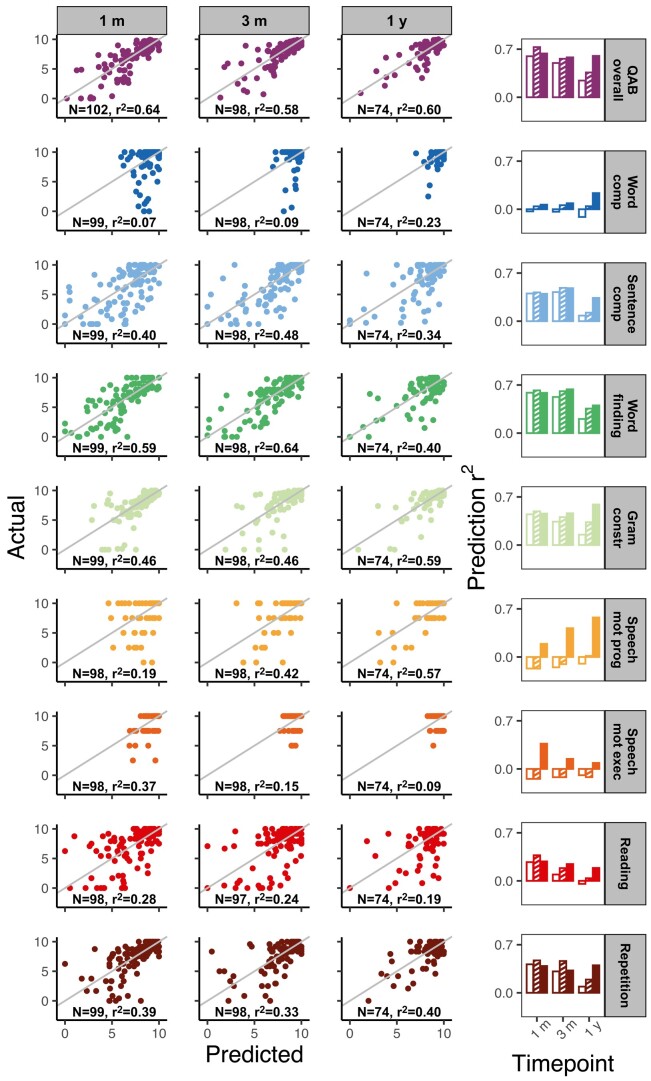
**Model performance for the lesion load + initial severity models.** As in Fig. 3 except that model performance reflects the inclusion of initial QAB overall score as an additional predictor. Note that acute predictions are not shown due to the presence of an acute score among the model predictors.

QAB overall was predicted with *r*^2^ = 0.64 at the 1-month timepoint, *r*^2^ = 0.58 at the 3-month timepoint and *r*^2^ = 0.60 at the 1-year timepoint (Fig. 4). (Note that prediction at the acute timepoint was not included because acute scores were among the model predictors.)

For QAB overall score, reduced models including only IP, demographic and stroke type information were already relatively predictive of outcomes at the 1-month timepoint; however, this predictive utility of the reduced models decreased notably at later timepoints. This contrasts with the full models, which either retained or increased their predictive utility as time post-stroke increased.

As above, predictive power varied for the nine QAB subscores (Fig. 4, Supplementary Table 2). Word finding, grammatical construction, speech motor programming and repetition were predicted particularly well across all timepoints, while single-word comprehension, speech motor execution and reading again proved more difficult to predict.

LLVs improved performance in 19 out of 27 (timepoints by subscores) cases, again most notably as time post-stroke increased. This pattern was particularly salient for the sentence comprehension, grammatical construction, reading and repetition subscores.

### Neural predictors of overall aphasia severity

**Figure 5:**
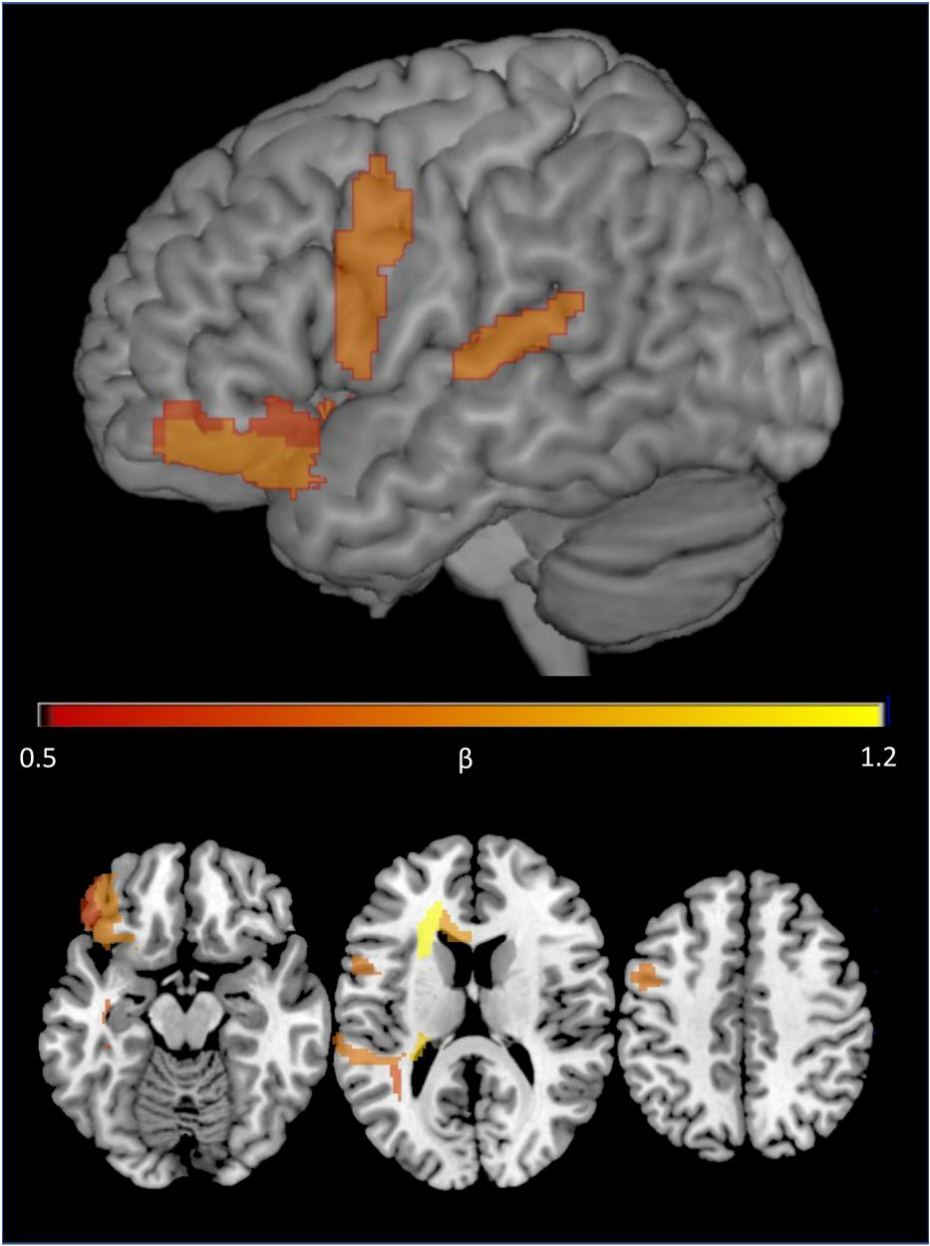
**Neural predictors of long-term aphasia severity.** Regions corresponding to negative feature weights 1 SD more extreme than the mean in the lesion load model, in which damage was predictive of lower overall QAB scores at the 1-year timepoint. Brighter regions correspond to beta weights associated with larger reductions in QAB overall at the 1-year timepoint.

In order to investigate which regions may be most associated with aphasia severity in the long term, we probed feature weights for the QAB overall predictions at the 1-year timepoint using the LLV model. Grey matter predictors of lower QAB scores included the left superior temporal gyrus (STG), precentral gyrus, orbital gyrus and basal ganglia; white matter predictors included the left anterior corona radiata, retrolenticular internal capsule, genu of the corpus callosum, sagittal stratum and superior longitudinal fasciculus (Fig. 5).

## Discussion

Our findings indicate that a great deal of the variance in long-term recovery from aphasia can be effectively predicted using SVR-based lesion–symptom mapping models and show that information about the location of a lesion, beyond simply its size, is in many cases crucial for making these predictions. This finding holds true even in cases where initial severity is accounted for, particularly at later timepoints post-stroke. Strengths of our study include its large and representative sample, its prospective longitudinal design, its detailed characterization of language using a validated aphasia battery and its careful consideration of best practices in multivariate lesion symptom mapping.^38^

This work is the first to our knowledge to systematically predict language outcomes for multiple predefined timepoints and on multiple language domains post-stroke. This work provides a quantitative follow-up to a recent descriptive study detailing trajectories of recovery from aphasia based on acute neuroimaging.^4^

Across models and timepoints, QAB overall and word finding were the outcomes that could be predicted most reliably, while outcomes in single-word comprehension, speech motor execution and reading were more difficult to predict. From a clinical perspective, QAB overall and word finding—often considered to be the ‘hallmark deficit’ of aphasia^32^—are particularly useful metrics to be able to forecast, due to their clear relationship to disability status and long-term independence for patients.^41-43^ The apparent lack of predictive ability for single-word comprehension and speech motor execution may reflect the fact that these deficits tend to resolve well in the long term,^4^ leaving minimal available variance for the models to learn from or predict. Reading, however, demonstrated poor prediction accuracy despite showing more variable outcomes in the long term. Future work should aim to investigate the ability to prognosticate reading outcomes in more detail using evaluations that more comprehensively account for various profiles of alexia with theoretically distinct anatomical bases.^44^

Including information about lesion location in the form of LLVs led to improvements in prediction accuracy in most models (34/36 LLV models, 19/27 LLV + IP models). While models that included acute QAB overall score sometimes performed well at the 1-month timepoint even without the inclusion of detailed lesion information, the addition of lesion load information regularly led to increases in predictive ability at the 3-month and 1-year timepoints. This pattern may reflect the complex nature of the acute post-stroke period, in which various factors not captured by our models, such as hypoperfusion, diaschisis and/or other medical complications, exert more influence, compared to later timepoints by which these issues have largely resolved and rendered lesion location a clearer predictor.

Our finding that lesion location–based predictions are more accurate at later timepoints is in line with prior work demonstrating transience and changeability in aphasia particularly in the early post-stroke period^4,45,46^; however, it stands in opposition to a theorized ‘proportional recovery rule’ stating that individuals with stroke tend to recover some fixed proportion of their lost function.^23,47^ It is important to note, however, that the original claims in these proportional recovery studies were limited by small sample sizes, and these findings have similarly been disputed from a statistical perspective, as the correlations that appear to support proportional recovery have been shown to occur even in simulated data with no true association between baseline and outcome scores.^27,48-50^ Thus, while initial language presentation may be a good predictor of outcomes in the short term, information about the integrity of specific anatomical regions may be more useful for effectively predicting outcomes in the chronic stage.

Regions in which damage was the most associated with greater aphasia severity in the long term fell in both grey and white matter: specifically, the left posterior STG, precentral gyrus, orbital gyrus, middle frontal gyrus and basal ganglia in grey matter and the anterior corona radiata, retrolenticular internal capsule, genu of the corpus callosum, sagittal stratum and superior longitudinal fasciculus in white matter. The left posterior STG has long been known to play a crucial role in language, though the particulars of its role and the anatomical bounds of the relevant region have been a subject of much debate^9-12,35,51-54^; the precentral gyrus is also a known language region associated with both phonological processing and speech motor programming.^55-57^ These regions are thus reasonably expected correlates of long-term impairment in language. However, the absence of ‘Broca’s area’ as a predictor of long-term impairment is noteworthy and is in line with prior work demonstrating that most aphasias following lesions to this region are transient in nature.^58-61^ Regarding white matter predictors, the extent to which grey versus white matter measures are valuable for prediction is disputed, with some researchers suggesting metrics of structural connectivity increase predictive accuracy^24,29^ and others claiming white matter information is largely redundant with grey matter measures.^27,28^ Prior work has, however, noted the importance of white matter ‘bottlenecks’ in left frontal and temporoparietal regions for supporting language function,^62-64^ which aligns with our finding that the top two strongest predictors of overall outcomes were in anterior regions of the corona radiata and posterior regions of the internal capsule, close to these proposed bottlenecks.

While this study is the first to our knowledge to specifically predict longitudinal language outcomes across multiple domains of language post-stroke, a handful of previous studies have used similar approaches to explore the extent to which post-stroke language abilities can be predicted using machine learning analyses of neuroimaging data. Most of these studies have been cross-sectional in nature, that is, investigating language performance in chronic cohorts at a single timepoint without reference to their acute presentation. Among these cross-sectional studies, some chose aphasia subtypes or global measures of aphasia severity as their outcomes of interest^25,26^; others predicted more specific measures but achieved only modest predictive accuracy in out-of-sample testing, e.g. *r*^2^ = 0.44–0.49,^24,29,30^ even as calculated using the squared correlation coefficient (a more liberal metric of predictive accuracy than prediction *r*^2^ reported here^39^). To our knowledge, the previous study most similar to the present study is Hope *et al*.,^14^ one of the only studies using multivariate lesion symptom mapping to make an explicit attempt to account for recovery. This study used Gaussian process regression based on structural imaging data and clinical variables to predict a measure of speech production derived from the Comprehensive Aphasia Test^3^ at both single and multiple timepoints. However, while this study had a large initial sample size of 270 total patients, only 38 individuals were assessed longitudinally, and these individuals varied widely in the times of assessment post-stroke. Additionally, the study focused only on speech production.

The ability to accurately predict aphasia outcomes as demonstrated herein could have a positive impact on clinical practice and individuals living with aphasia. First, a better baseline understanding of expected trajectories of recovery from aphasia lays the groundwork for assessing the efficacy of treatment in clinical practice and/or clinical trials. Second, the ability to provide a patient with a sense of what recovery is likely to look like ‘for them’, specifically, could help to set realistic expectations for the patient, their loved ones and their clinical team, such that appropriate strategies for managing impairment and collaborative goal setting could be put into place.^65^ Finally, while speech-language pathologists tend to recognize the importance of neuroanatomical awareness in clinical practice,^66,67^ neuroanatomical information is often found intimidating^68^ and can be poorly retained.^69^ Thus, developing algorithms which can help to ‘interpret’ neuroimaging data, using technology similar to the models described here, may help clinicians across the spectrum of care more easily make neuroanatomically informed predictions for patients.

Regarding the real-world applicability of using neuroimaging to predict language recovery, Shuster^70^ has raised concerns about prior attempts at this aim, citing, for example, a lack of regard for individual differences, poor validation on independent data sets, inaccessibility of scanner environments for certain patients and inattention to predictors that do not relate directly to the academic hypotheses in question. We have addressed many of these concerns in the present study: individual differences are accounted for via the positioning of patients in a multidimensional symptom space; leave-one-out cross-validation helps to handle the risk of overfitting; patients who were not MRI safe are included via drawing lesions on CTs; demographic and non–lesion-based predictors are already included, with even more predictors planned for inclusion in the future. Nevertheless, this work should simply be considered an early step towards a better understanding of the myriad factors that can influence language recovery, considered in tandem with other individual patient characteristics, therapeutic interventions and changes in neural function due to neuroplasticity. Indeed, machine learning approaches are simply models and should always be considered as a supplement to, rather than a replacement for, clinical expertise.

### Limitations

This study has several notable limitations. First, many of the limitations noted in Wilson *et al*.^4^ remain relevant to this follow-up study. As noted therein, the QAB is designed to be brief and therefore cannot comprehensively account for all aspects of language and associated functions; lesions were delineated using only acute neuroimaging, which may not be entirely reflective of irreversible neural damage; and sample sizes decreased longitudinally, with smaller sample sizes at later timepoints. Though there were no differences in severity across patients with and without follow-up timepoints, future studies with larger sample sizes at later timepoints will be necessary to verify the findings reported here.

Second, we chose to use within-sample leave-one-out cross-validation to assess the predictive accuracy of our models. Although the training and validation data used in our cross-validation procedure were fully independent, we were not able to hold out a truly independent test set to evaluate final model performance without sacrificing the power of our sample size. As data from future patients is collected, this new data will become the test set upon which the true generalizability of our models can be assessed. Prior work has discussed potential pitfalls of leave-one-out cross-validation, in particular the potential for anti-correlation between training and testing data in the presence of high variance across test exemplars.^71^ However, it is important to note that there are trade-offs incurred by all methods of cross-validation.^38,72^ Given our relatively small sample sizes at later timepoints and the relatively consistent lesion–symptom relationships observed, the bias–variance trade-offs incurred by using leave-one-out cross-validation were deemed preferable to those associated with holding out larger testing sets, especially decreased power to detect relationships between predictors and language symptoms. This issue could, again, be addressed in future studies with larger samples.

Finally, the reporting of neural correlates of language outcomes using beta weights to ascribe importance to particular spatial predictors of aphasia severity is experimental. The interpretation of feature weights in machine learning models, even in linear models as used here, is not straightforward due to the fact that they are calculated to meet algorithm-specific regularization constraints, rather than to model a direct relationship with the behavioural variable in question.^40,73,74^ Thus, these results should be interpreted with caution.

## Conclusion

This study is the first to systematically predict language outcomes for multiple predefined timepoints and on multiple speech and language domains post-stroke, explaining about three-fifths of the variance in aphasia outcome at 1 year. Our findings demonstrate that information about lesion location is crucial for making many of these predictions, particularly at later timepoints post-stroke. This work both demonstrates the feasibility of using SVR models to make precise and personalized predictions about long-term recovery from aphasia and provides a valuable structural baseline upon which to build more elaborate models, including information about functional language organization, brain health, diffusion tractography and/or speech and language therapy. Such models could help to further clarify what is different when, structural damage being equal, recovery is more successful in some individuals than others. Taken together, these scientific endeavours will aid both clinicians and scientists by providing a more effective means to predict outcomes in aphasia and by further elucidating the neural bases of language.

## Supplementary Material

fcae024_Supplementary_Data

## Data Availability

Preprocessed data and the code for calculating all values reported herein are available at https://github.com/dflevy/mlsm_brainComms/. An interactive website including all data is forthcoming and will be made available in the future at https://langneurosci.org/recovery.

## References

[1] Kertesz A, McCabe P. Recovery patterns and prognosis in aphasia. Brain. 1977;100(1):1–18. 861709 10.1093/brain/100.1.1

[2] Pedersen PM, Jørgensen HS, Nakayama H, Raaschou HO, Olsen TS. Aphasia in acute stroke: Incidence, determinants, and recovery. Ann Neurol. 1995;38(4):659–666. 7574464 10.1002/ana.410380416

[3] Swinburn K, Porter G, Howard D. Comprehensive aphasia test. Psychology Press; 2004.

[4] Wilson SM, Entrup JL, Schneck SM, et al Recovery from aphasia in the first year after stroke. Brain. 2023;146(3):1021–1039. 35388420 10.1093/brain/awac129 PMC10169426

[5] Laska AC, Hellblom A, Murray V, Kahan T, Von Arbin M. Aphasia in acute stroke and relation to outcome. J Intern Med. 2001;249(5):413–422. 11350565 10.1046/j.1365-2796.2001.00812.x

[6] Pedersen PM, Vinter K, Olsen TS. Aphasia after stroke: Type, severity and prognosis. Cerebrovasc Dis Basel Switz. 2004;17(1):35–43. 10.1159/000073896 14530636

[7] Wilson SM, Eriksson DK, Brandt TH, et al Patterns of recovery from aphasia in the first 2 weeks after stroke. J Speech Lang Hear Res. 2019;62(3):723–732. 30950735 10.1044/2018_JSLHR-L-18-0254 PMC6802900

[8] Kertesz A, Harlock W, Coates R. Computer tomographic localization, lesion size, and prognosis in aphasia and nonverbal impairment. Brain Lang. 1979;8(1):34–50. 476474 10.1016/0093-934x(79)90038-5

[9] Selnes OA, Knopman DS, Niccum N, Rubens AB, Larson D. Computed tomographic scan correlates of auditory comprehension deficits in aphasia: A prospective recovery study. Ann Neurol. 1983;13(5):558–566. 6870207 10.1002/ana.410130515

[10] Selnes OA, Niccum N, Knopman DS, Rubens AB. Recovery of single word comprehension: CT-scan correlates. Brain Lang. 1984;21(1):72–84. 6199078 10.1016/0093-934x(84)90037-3

[11] Naeser MA, Helm-Estabrooks N, Haas G, Auerbach S, Srinivasan M. Relationship between lesion extent in “Wernicke’s area” on computed tomographic scan and predicting recovery of comprehension in Wernicke’s aphasia. Arch Neurol. 1987;44(1):73–82. 3800725 10.1001/archneur.1987.00520130057018

[12] Kertesz A, Lau WK, Polk M. The structural determinants of recovery in Wernicke’s aphasia. Brain Lang. 1993;44(2):153–164. 8428309 10.1006/brln.1993.1010

[13] Goldenberg G, Spatt J. Influence of size and site of cerebral lesions on spontaneous recovery of aphasia and on success of language therapy. Brain Lang. 1994;47(4):684–698. 7859059 10.1006/brln.1994.1063

[14] Hope TMH, Seghier ML, Leff AP, Price CJ. Predicting outcome and recovery after stroke with lesions extracted from MRI images. NeuroImage Clin. 2013;2:424–433. 24179796 10.1016/j.nicl.2013.03.005 PMC3778268

[15] Ramsey LE, Siegel JS, Lang CE, Strube M, Shulman GL, Corbetta M. Behavioural clusters and predictors of performance during recovery from stroke. Nat Hum Behav. 2017;1:0038. 28713861 10.1038/s41562-016-0038 PMC5508212

[16] Hillis AE, Beh YY, Sebastian R, et al Predicting recovery in acute poststroke aphasia. Ann Neurol. 2018;83(3):612–622. 29451321 10.1002/ana.25184 PMC5867273

[17] Benghanem S, Rosso C, Arbizu C, et al Aphasia outcome: The interactions between initial severity, lesion size and location. J Neurol. 2019;266(6):1303–1309. 30820740 10.1007/s00415-019-09259-3

[18] Nakagawa Y, Sano Y, Funayama M, Kato M. Prognostic factors for long-term improvement from stroke-related aphasia with adequate linguistic rehabilitation. Neurol Sci Off J Ital Neurol Soc Ital Soc Clin Neurophysiol. 2019;40(10):2141–2146. 10.1007/s10072-019-03956-7 PMC6745027 31183673

[19] Basso A, Lecours AR, Moraschini S, Vanier M. Anatomoclinical correlations of the aphasias as defined through computerized tomography: Exceptions. Brain Lang. 1985;26(2):201–229. 2417656 10.1016/0093-934x(85)90039-2

[20] Plowman E, Hentz B, Ellis C. Post-stroke aphasia prognosis: A review of patient-related and stroke-related factors: Aphasia prognosis. J Eval Clin Pract. 2012;18(3):689–694. 21395923 10.1111/j.1365-2753.2011.01650.x

[21] Watila MM, Balarabe SA. Factors predicting post-stroke aphasia recovery. J Neurol Sci. 2015;352(1–2):12–18. 25888529 10.1016/j.jns.2015.03.020

[22] Gerstenecker A, Lazar RM. Language recovery following stroke. Clin Neuropsychol. 2019;33(5):928–947. 30698070 10.1080/13854046.2018.1562093 PMC8985654

[23] Lazar RM, Minzer B, Antoniello D, Festa JR, Krakauer JW, Marshall RS. Improvement in aphasia scores after stroke is well predicted by initial severity. Stroke. 2010;41(7):1485–1488. 20538700 10.1161/STROKEAHA.109.577338 PMC2921806

[24] Yourganov G, Fridriksson J, Rorden C, Gleichgerrcht E, Bonilha L. Multivariate connectome-based symptom mapping in post-stroke patients: Networks supporting language and speech. J Neurosci. 2016;36(25):6668–6679. 27335399 10.1523/JNEUROSCI.4396-15.2016 PMC4916245

[25] Yourganov G, Smith KG, Fridriksson J, Rorden C. Predicting aphasia type from brain damage measured with structural MRI. Cortex J Devoted Study Nerv Syst Behav. 2015;73:203–215. 10.1016/j.cortex.2015.09.005 PMC4689665 26465238

[26] Del Gaizo J, Fridriksson J, Yourganov G, et al Mapping language networks using the structural and dynamic brain connectomes. eNeuro. 2017;4(5):ENEURO.0204-17.2017. 10.1523/ENEURO.0204-17.2017 PMC5672546 29109969

[27] Hope TMH, Leff AP, Price CJ. Predicting language outcomes after stroke: Is structural disconnection a useful predictor? NeuroImage Clin. 2018;19:22–29. 30034998 10.1016/j.nicl.2018.03.037 PMC6051761

[28] Halai AD, Woollams AM, Lambon Ralph MA. Investigating the effect of changing parameters when building prediction models for post-stroke aphasia. Nat Hum Behav. 2020;4(7):725–735. 32313234 10.1038/s41562-020-0854-5 PMC7116235

[29] Kristinsson S, Zhang W, Rorden C, et al Machine learning-based multimodal prediction of language outcomes in chronic aphasia. Hum Brain Mapp. 2021;42(6):1682–1698. 33377592 10.1002/hbm.25321 PMC7978124

[30] Pustina D, Coslett HB, Ungar L, et al Enhanced estimations of post-stroke aphasia severity using stacked multimodal predictions. Hum Brain Mapp. 2017;38(11):5603–5615. 28782862 10.1002/hbm.23752 PMC5765865

[31] Wilson SM, Eriksson DK, Schneck SM, Lucanie JM. A quick aphasia battery for efficient, reliable, and multidimensional assessment of language function. PLoS One. 2018;13(2):e0192773. 29425241 10.1371/journal.pone.0192773 PMC5806902

[32] Bonilha L, Gleichgerrcht E, Nesland T, Rorden C, Fridriksson J. Success of anomia treatment in aphasia is associated with preserved architecture of global and left temporal lobe structural networks. Neurorehabil Neural Repair. 2016;30(3):266–279. 26150147 10.1177/1545968315593808 PMC4703576

[33] Mori S, Wakana S, van Zijl PCM, Nagae-Poetscher LM. MRI atlas of human white matter. Elsevier; 2005. 10.1148/radiol.2301021640 14645885

[34] Fan L, Li H, Zhuo J, et al The human brainnetome atlas: A new brain atlas based on connectional architecture. Cereb Cortex N Y N 1991. 2016;26(8):3508–3526. 10.1093/cercor/bhw157 PMC4961028 27230218

[35] Wilson SM, Bautista A, McCarron A. Convergence of spoken and written language processing in the superior temporal sulcus. NeuroImage. 2018;171:62–74. 29277646 10.1016/j.neuroimage.2017.12.068 PMC5857434

[36] Vapnik VN . Statistical learning theory. Wiley; 1998. 10.1109/72.788640 18252602

[37] Zhang Y, Kimberg DY, Coslett HB, Schwartz MF, Wang Z. Multivariate lesion-symptom mapping using support vector regression. Hum Brain Mapp. 2014;35(12):5861–5876. 25044213 10.1002/hbm.22590 PMC4213345

[38] Scheinost D, Noble S, Horien C, et al Ten simple rules for predictive modeling of individual differences in neuroimaging. NeuroImage. 2019;193:35–45. 30831310 10.1016/j.neuroimage.2019.02.057 PMC6521850

[39] Alexander DLJ, Tropsha A, Winkler DA. Beware of R2: Simple, unambiguous assessment of the prediction accuracy of QSAR and QSPR models. J Chem Inf Model. 2015;55(7):1316–1322. 26099013 10.1021/acs.jcim.5b00206 PMC4530125

[40] Kriegeskorte N, Douglas PK. Interpreting encoding and decoding models. Curr Opin Neurobiol. 2019;55:167–179. 31039527 10.1016/j.conb.2019.04.002 PMC6705607

[41] Spaccavento S, Craca A, Del Prete M, et al Quality of life measurement and outcome in aphasia. Neuropsychiatr Dis Treat. 2013;10:27–37. 24368886 10.2147/NDT.S52357 PMC3869916

[42] Hilari K, Needle JJ, Harrison KL. What are the important factors in health-related quality of life for people with aphasia? A systematic review. Arch Phys Med Rehabil. 2012;93(1, Supplement):S86–S95.e4. 22119074 10.1016/j.apmr.2011.05.028

[43] Best W, Greenwood A, Grassly J, Hickin J. Bridging the gap: Can impairment-based therapy for anomia have an impact at the psycho-social level? Int J Lang Commun Disord. 2008;43(4):390–407. 18584417 10.1080/13682820701608001

[44] Cloutman LL, Newhart M, Davis CL, Heidler-Gary J, Hillis AE. Neuroanatomical correlates of oral reading in acute left hemispheric stroke. Brain Lang. 2011;116(1):14–21. 20889196 10.1016/j.bandl.2010.09.002 PMC2991537

[45] Pashek GV, Holland AL. Evolution of aphasia in the first year post-onset. Cortex J Devoted Study Nerv Syst Behav. 1988;24(3):411–423. 10.1016/s0010-9452(88)80004-2 3191724

[46] Bates E, Saygin AP, Moineau S, Marangolo P, Pizzamiglio L. Analyzing aphasia data in a multidimensional symptom space. Brain Lang. 2005;92(2):106–116. 15629486 10.1016/j.bandl.2004.06.108

[47] Marchi NA, Ptak R, Di Pietro M, Schnider A, Guggisberg AG. Principles of proportional recovery after stroke generalize to neglect and aphasia. Eur J Neurol. 2017;24(8):1084–1087. 28585297 10.1111/ene.13296

[48] Hawe RL, Scott SH, Dukelow SP. Taking proportional out of stroke recovery. Stroke. 2019;50(1):204–211. 30580742 10.1161/STROKEAHA.118.023006

[49] Bonkhoff AK, Hope T, Bzdok D, et al Bringing proportional recovery into proportion: Bayesian modelling of post-stroke motor impairment. Brain. 2020;143(7):2189–2206. 32601678 10.1093/brain/awaa146

[50] Bowman H, Bonkhoff A, Hope T, Grefkes C, Price C. Inflated estimates of proportional recovery from stroke. Stroke. 2021;52(5):1915–1920. 33827246 10.1161/STROKEAHA.120.033031 PMC7610699

[51] Wernicke C . Some new studies on aphasia (1886). In: Eling P, ed. Reader in the history of aphasia: From Franz Gall to Norman Geschwind. John Benjamins Publishing; 1994:90–96.

[52] Tremblay P, Dick A. Broca and Wernicke are dead, or moving past the classic model of language neurobiology. Brain Lang. 2016;162:60–71. 27584714 10.1016/j.bandl.2016.08.004

[53] Bogen JE, Bogen GM. Wernicke’s region—where is it? Ann N Y Acad Sci. 1976;280:834–843. 1070943 10.1111/j.1749-6632.1976.tb25546.x

[54] Binder JR . The Wernicke area: Modern evidence and a reinterpretation. Neurology. 2015;85(24):2170–2175. 26567270 10.1212/WNL.0000000000002219 PMC4691684

[55] Yen M, DeMarco AT, Wilson SM. Adaptive paradigms for mapping phonological regions in individual participants. NeuroImage. 2019;189:368–379. 30665008 10.1016/j.neuroimage.2019.01.040 PMC6424113

[56] Silva AB, Liu JR, Zhao L, Levy DF, Scott TL, Chang EF. A neurosurgical functional dissection of the middle precentral gyrus during speech production. J Neurosci. 2022;42(45):8416–8426. 36351829 10.1523/JNEUROSCI.1614-22.2022 PMC9665919

[57] Hickok G, Venezia J, Teghipco A. Beyond broca: Neural architecture and evolution of a dual motor speech coordination system. Brain. 2023;146(5):1775–1790. 36746488 10.1093/brain/awac454 PMC10411947

[58] Andrews JP, Cahn N, Speidel BA, et al Dissociation of Broca’s area from Broca’s aphasia in patients undergoing neurosurgical resections. J Neurosurg. 2022;1(aop):1–11. 10.3171/2022.6.JNS2297 PMC9899289 35932264

[59] Mohr JP, Pessin MS, Finkelstein S, Funkenstein HH, Duncan GW, Davis KR. Broca aphasia: Pathologic and clinical. Neurology. 1978;28(4):311–311. 565019 10.1212/wnl.28.4.311

[60] Fridriksson J, Fillmore P, Guo D, Rorden C. Chronic Broca’s aphasia is caused by damage to Broca’s and Wernicke’s areas. Cereb Cortex N Y NY. 2015;25(12):4689–4696. 10.1093/cercor/bhu152 PMC4669036 25016386

[61] Gajardo-Vidal A, Lorca-Puls DL, Team P, et al Damage to Broca’s area does not contribute to long-term speech production outcome after stroke. Brain. 2021;144(3):817–832. 33517378 10.1093/brain/awaa460 PMC8041045

[62] Bürgel U, Amunts K, Hoemke L, Mohlberg H, Gilsbach JM, Zilles K. White matter fiber tracts of the human brain: Three-dimensional mapping at microscopic resolution, topography and intersubject variability. NeuroImage. 2006;29(4):1092–1105. 16236527 10.1016/j.neuroimage.2005.08.040

[63] Turken AU, Dronkers NF. The neural architecture of the language comprehension network: Converging evidence from lesion and connectivity analyses. Front Syst Neurosci. 2011;5:1. 21347218 10.3389/fnsys.2011.00001 PMC3039157

[64] Griffis JC, Nenert R, Allendorfer JB, Szaflarski JP. Damage to white matter bottlenecks contributes to language impairments after left hemispheric stroke. NeuroImage Clin. 2017;14:552–565. 28337410 10.1016/j.nicl.2017.02.019 PMC5350568

[65] Haley KL, Cunningham KT, Barry J, de Riesthal M. Collaborative goals for communicative life participation in aphasia: The FOURC model. Am J Speech Lang Pathol. 2019;28(1):1–13. 31072164 10.1044/2018_AJSLP-18-0163

[66] Martin K, Bessell NJ, Scholten I. The perceived importance of anatomy and neuroanatomy in the practice of speech-language pathology. Anat Sci Educ. 2014;7(1):28–37. 23775941 10.1002/ase.1377

[67] Barros MD, Silva VA, Liquidato BM. Is anatomy important for speech language pathology (SLP) undergraduate students? FASEB J. 2017;31(1):732.14–732.14.27811061

[68] Javaid MA, Chakraborty S, Cryan JF, Schellekens H, Toulouse A. Understanding neurophobia: Reasons behind impaired understanding and learning of neuroanatomy in cross-disciplinary healthcare students: Anatomical sciences education. Anat Sci Educ. 2018;11(1):81–93. 28628732 10.1002/ase.1711

[69] Barros MD, Silva VA, Mendes CJL, Liquidato BM. Retention of anatomic knowledge in speech-language pathology undergraduate students. FASEB J. 2018;32(S1):508.1.

[70] Shuster LI . Considerations for the use of neuroimaging technologies for predicting recovery of speech and language in aphasia. Am J Speech Lang Pathol. 2018;27(1S):291–305. 29497745 10.1044/2018_AJSLP-16-0180

[71] Poldrack RA, Huckins G, Varoquaux G. Establishment of best practices for evidence for prediction: A review. JAMA Psychiatry. 2020;77(5):534–540. 31774490 10.1001/jamapsychiatry.2019.3671 PMC7250718

[72] Bengio Y, Grandvalet Y. No unbiased estimator of the variance of K-fold cross-validation. J Mach Learn Res. 2003;5:1089–1105.

[73] Haufe S, Meinecke F, Görgen K, et al On the interpretation of weight vectors of linear models in multivariate neuroimaging. NeuroImage. 2014;87:96–110. 24239590 10.1016/j.neuroimage.2013.10.067

[74] Sperber C, Wiesen D, Karnath HO. An empirical evaluation of multivariate lesion behaviour mapping using support vector regression. Hum Brain Mapp. 2019;40(5):1381–1390. 30549154 10.1002/hbm.24476 PMC6865618

