# Correction to: Multivariate lesion symptom mapping for predicting trajectories of recovery from aphasia

**DOI:** 10.1093/braincomms/fcae085

**Published:** 2024-03-26

This is a correction to: Deborah F Levy, Jillian L Entrup, Sarah M Schneck, Caitlin F Onuscheck, Maysaa Rahman, Anna Kasdan, Marianne Casilio, Emma Willey, L Taylor Davis, Michael de Riesthal, Howard S Kirshner, Stephen M Wilson, Multivariate lesion symptom mapping for predicting trajectories of recovery from aphasia, *Brain Communications*, Volume 6, Issue 1, 2024, fcae024, https://doi.org/10.1093/braincomms/fcae024

In the originally published version of the article, a citation was misnumbered in the caption of Figure 1. The caption has been corrected accordingly.

