# Supplementary material for: Multivariate lesion symptom mapping for predicting trajectories of recovery from aphasia
Source: Brain Commun. 2024 Feb 1;6(1):fcae024. doi: 10.1093/braincomms/fcae024 (PMC10873140; doi:10.1093/braincomms/fcae024)
Supplement: fcae024_Supplementary_Data [file fcae024_supplementary_data.pdf]

Using math and the brain to predict language recovery after stroke

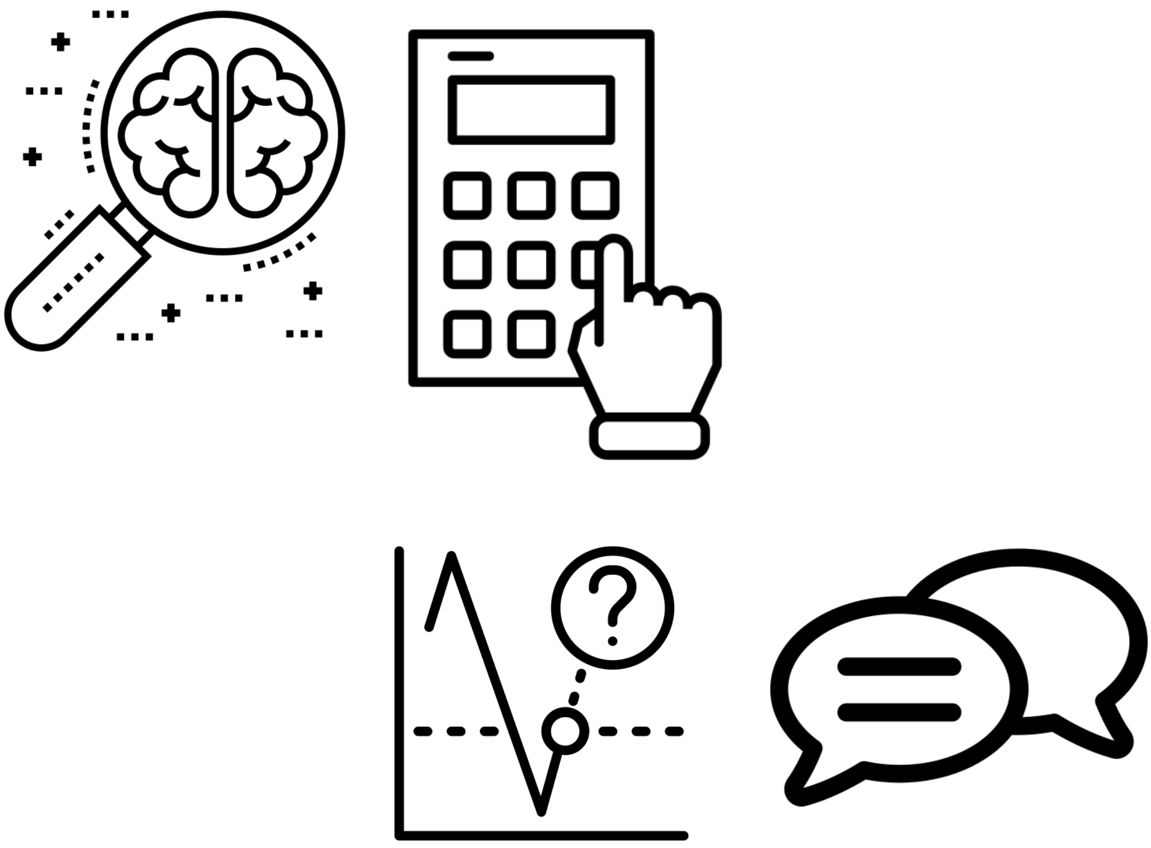

Supplementary Figure 1: Aphasia-friendly manuscript.
An accessible version of "Multivariate lesion-symptom mapping for predicting trajectories of recovery from aphasia" designed for individuals with aphasia and their loved ones.

Abstract / Summary

- **Aphasia** is a **problem** with **language** that can happen after **stroke**

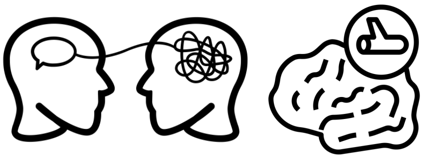

- Language usually **gets better**, but we can't always **predict how much**

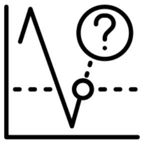

- We looked at a **big group** of **people** with **aphasia**, their **brains**, and their **language**

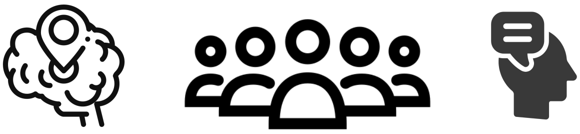

- We used **math** to try and **predict language** across the **first year** after stroke

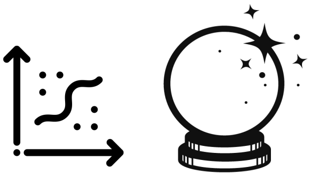

- This math did a **pretty good job** making predictions (about **60%** correct)!

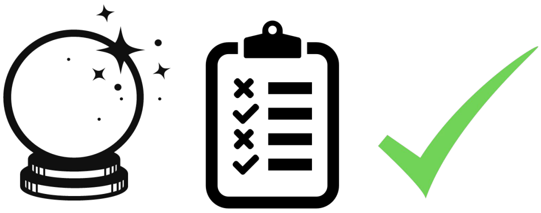

- We **hope** that more math like this will **help** doctors, therapists, researchers, and people with aphasia have **clearer expectations** about **aphasia recovery**

Introduction (1):
What we know about aphasia recovery

- **Aphasia** is a **problem** with **language** that can happen after **stroke** — usually on the **left** side of the **brain**

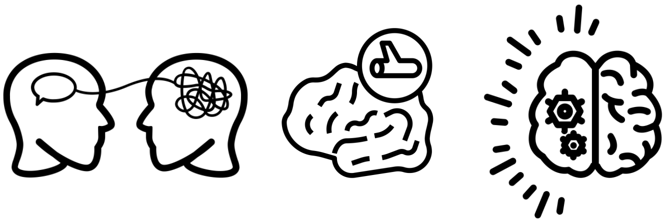

- Aphasia almost always **gets better**, but we **can't** always **predict how much**

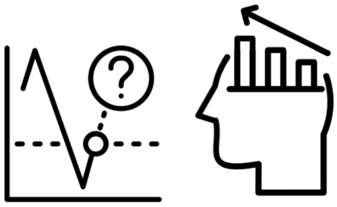

- Strokes can happen in different **parts** of the **brain**

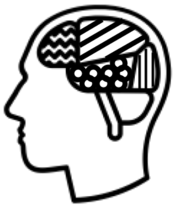

- Usually **where** a stroke happened **tells** us the **most** about **language** over **time**

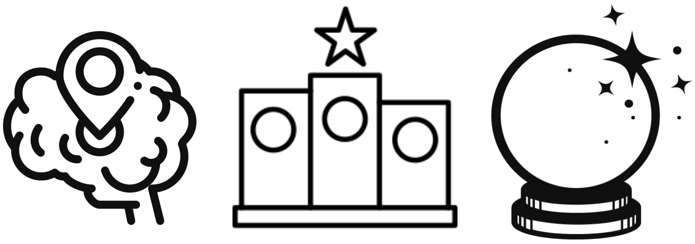

Introduction (2): Why we want to know more

- It would be **good** to be able to **predict language** after **stroke** for lots of people, like… 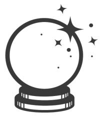
    - **People** with **aphasia** (to know what to **expect**)

      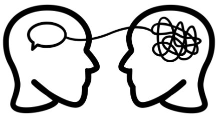
    - Their **family** and **friends** (to help **support** and **plan**)

      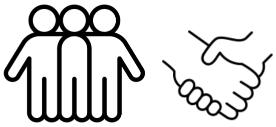
    - **Doctors** and **therapists** (to help **inform** and **treat** their **patients**)

      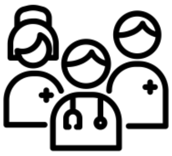
    - **Researchers** (to better **understand** language and the brain)

      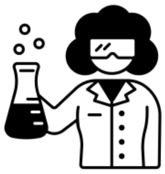
- Other **scientists** have **tried** this **before**, but we had some **new ideas** about how to do it!

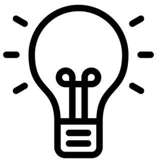

Introduction (3):
What we did in this study

- In this study, we use a special type of **math** (called *SVR*) to take things we **know** about people **right after stroke**, like…

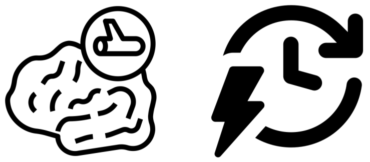

  - Their **age** and **education**

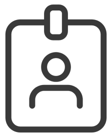

  - Their **brain scans**

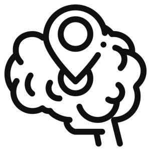

  - Their **language** abilities

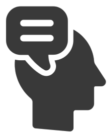

- …and **predict** what **language** will be like at **later** times **after stroke**

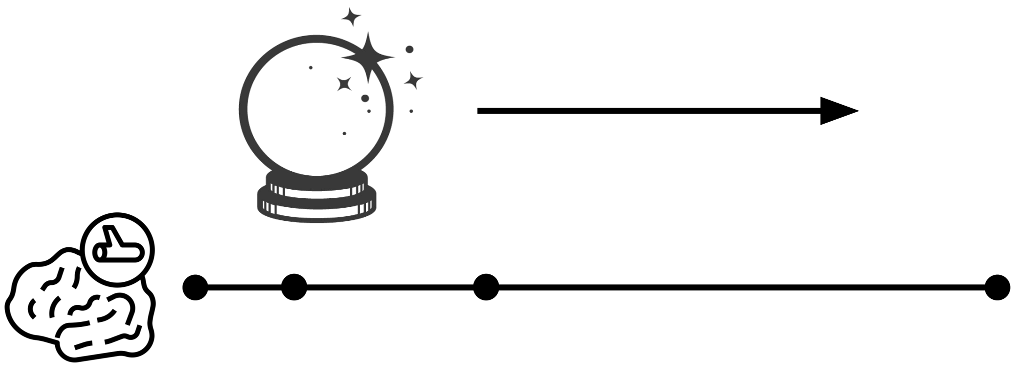

Methods (1):
Getting data from right after a stroke

- We met **217 people** with **aphasia** a few **days after** a **stroke**

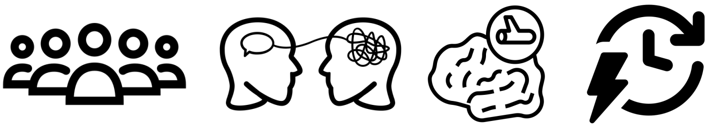

- We got **permission** to include them in this study

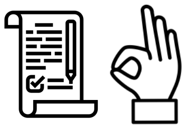

- We **tested** their **language** using a test called the **Quick Aphasia Battery (QAB)**

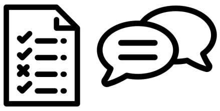

- We used a **computer program** to **highlight** the **stroke** on pictures of their **brains**

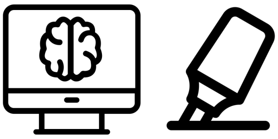

- We wrote code to **count** how much **damage** was in **different parts** of the **brain**

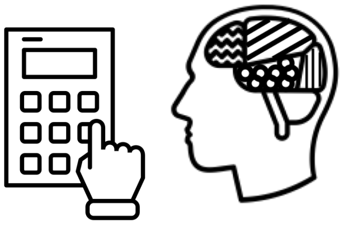

Methods (2):
Getting more data later on

- We tried to **follow up** with each person **3 more times**…
    - **1 month** after their stroke

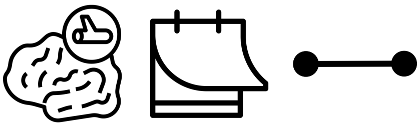

    - **3 months** after their stroke

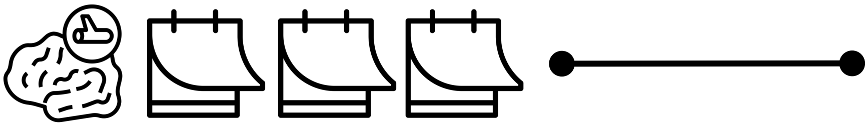

    - **1 year** after their stroke

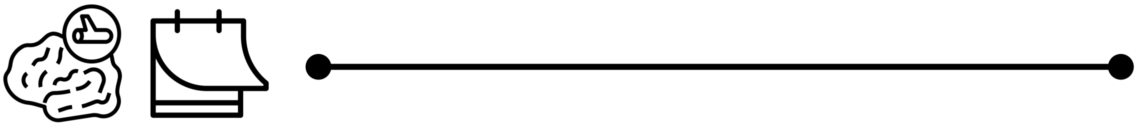

- …and give them the **same** language **test** (the **QAB**)

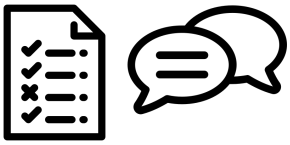

Methods (3):
Using math to make predictions

- We used a **special** kind of **math** (called *SVR*) to predict language **scores** based on each **person** and their **brain**

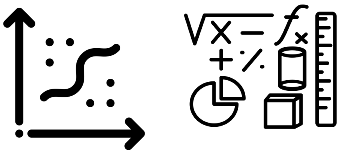

- We did this **2 DIFFERENT WAYS**:
  - **NOT USING** information about the person's **language** right after stroke

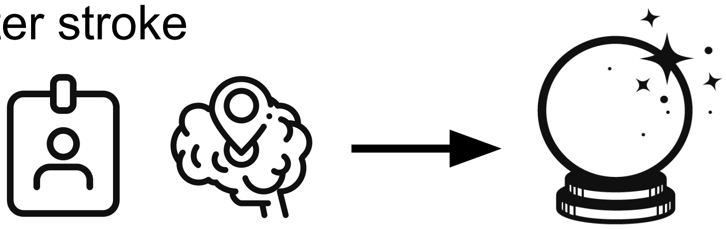

  - **USING** information about the person's **language** right after stroke

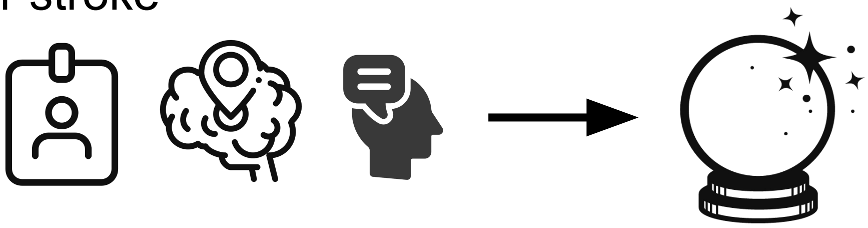

- We then **compared** each **prediction** to each person's **real language score** to see **how well** we did!

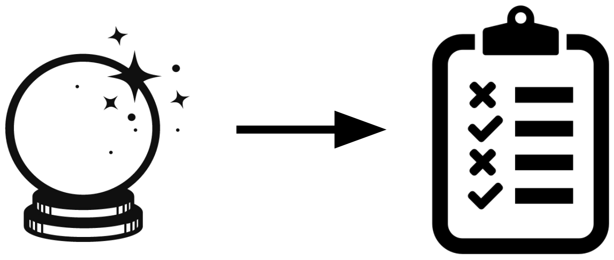

Results (1): Predicting from person and brain data only

- When we **ONLY** used data on each **person** and their **brain** (**NOT** on their **language**), we did a **pretty good job** predicting overall language

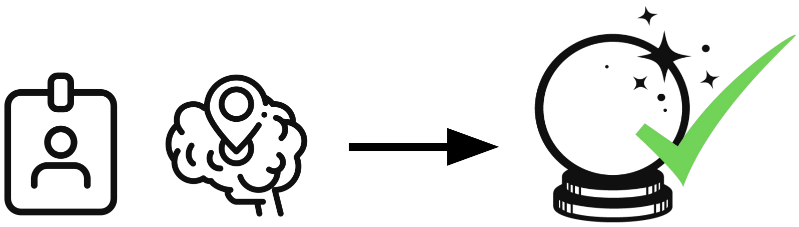

- We did differently at different times…
  - Around **38%** accurate **right after** stroke
  - Around **41%** accurate **1 month** after stroke
  - Around **46%** accurate **3 months** after stroke
  - Around **59%** accurate **1 year** after stroke

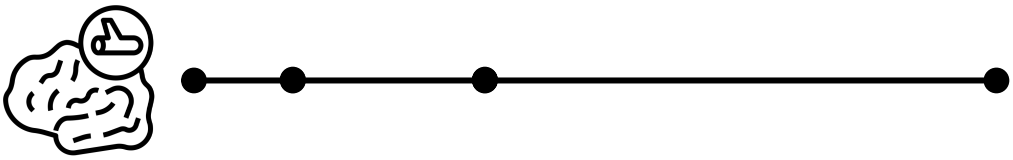

- …but information about the **brain** was **important** at all of them

Results (2): Predicting from person, brain, and language data

- When we **ALSO** used information about **language** right after stroke (**plus** information on the **person** and their **brain**), we (again) did a **pretty good job**!

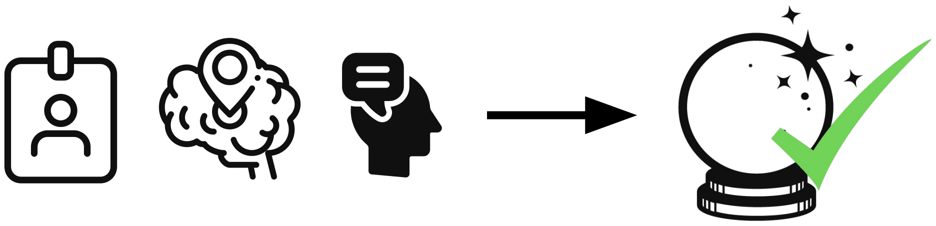

- We (again) did differently at different times…
  - Around **64%** accurate **1 month** after stroke
  - Around **58%** accurate **3 months** after stroke
  - Around **60%** accurate **1 year** after stroke

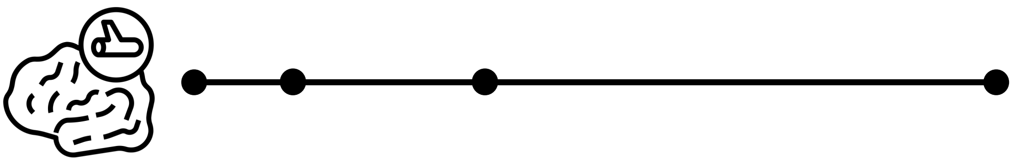

- …but information about the **brain** was (still!) **important** at all of them

Results (3):
What parts of the brain matter most?

- Certain **parts** of the **brain** helped us the **most** to make good predictions about language 1 year after stroke

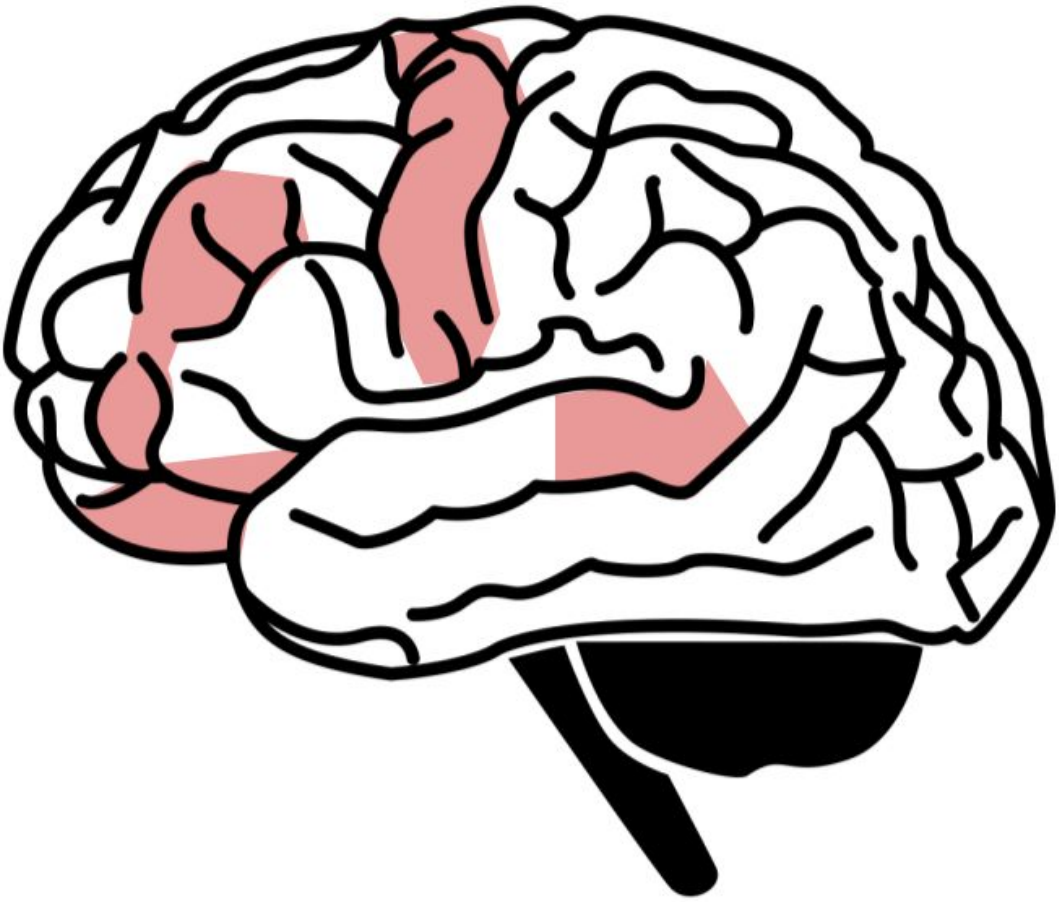

Discussion (1):
Main ideas

- We think this is the **first study** to **predict language recovery** over **time** in this way

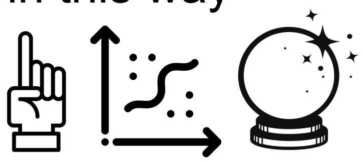

- It was **easier** to predict certain **parts** of **language**, like coming up with words

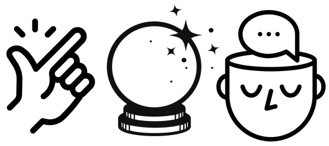

- It was **harder** to **predict** other **parts** of **language**, like reading and understanding words

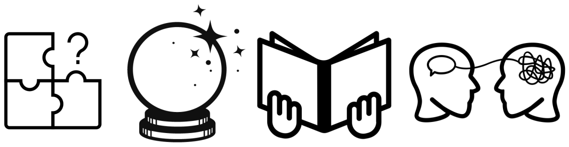

- Using information about the **brain** always helped us make **better predictions** in all cases

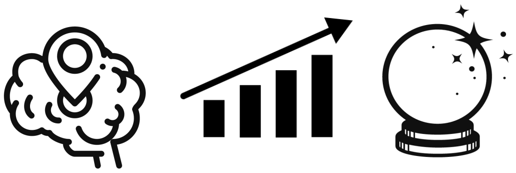

Discussion (2):
Why this matters

- What we found both **makes sense with** and **expands on** older studies

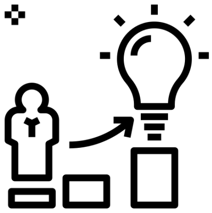

- We think our study is **important** because it will **help** people know what to **expect** in **aphasia recovery**

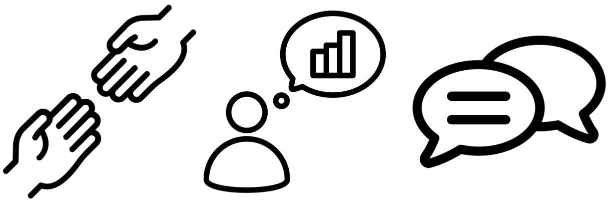

- We tried our best to do a really **thorough** job!

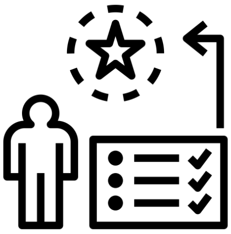

Discussion (3):
Limitations / things to think about

- The **QAB language test** is **short** and **doesn't cover everything** there is to know about language

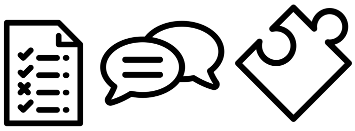

- How a **stroke** looks on a brain image **can change** over **time**

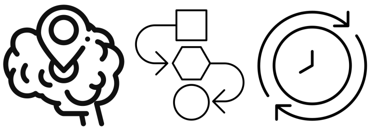

- **Not everyone** we met right after stroke **came back** for a **follow up** language test

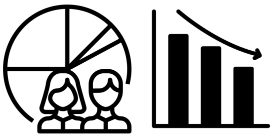

- Some **scientists** do this **math** in slightly **different ways**, so might get slightly different results

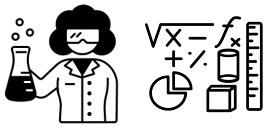

- There is still **a lot to learn** about what this kind of **math** can tell us about specific parts of the **brain**

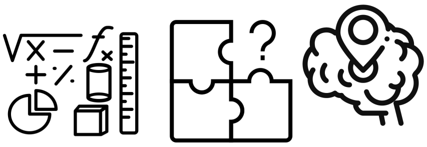

Conclusion

- This is the **first study** to make **predictions** about **language recovery** across the first **year** of **aphasia** after stroke using data from the **brain**

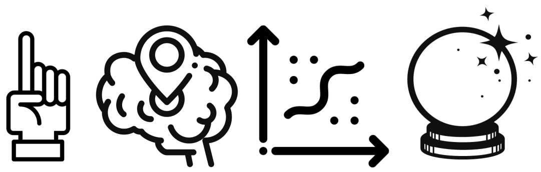

- We learned that knowing about the **brain** can tell us a lot (about **60%**) of what people can **expect** in recovery from **aphasia**!

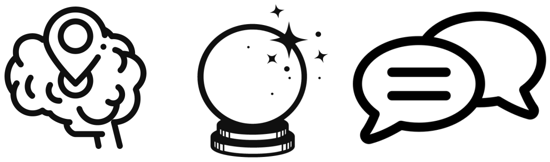

- **This** information (**plus more** we are thinking about!) could **help** lots of people to have **clearer expectations** about recovery

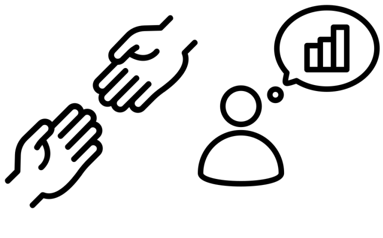

- We plan to write **more studies** about this **soon**!

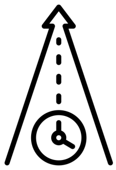

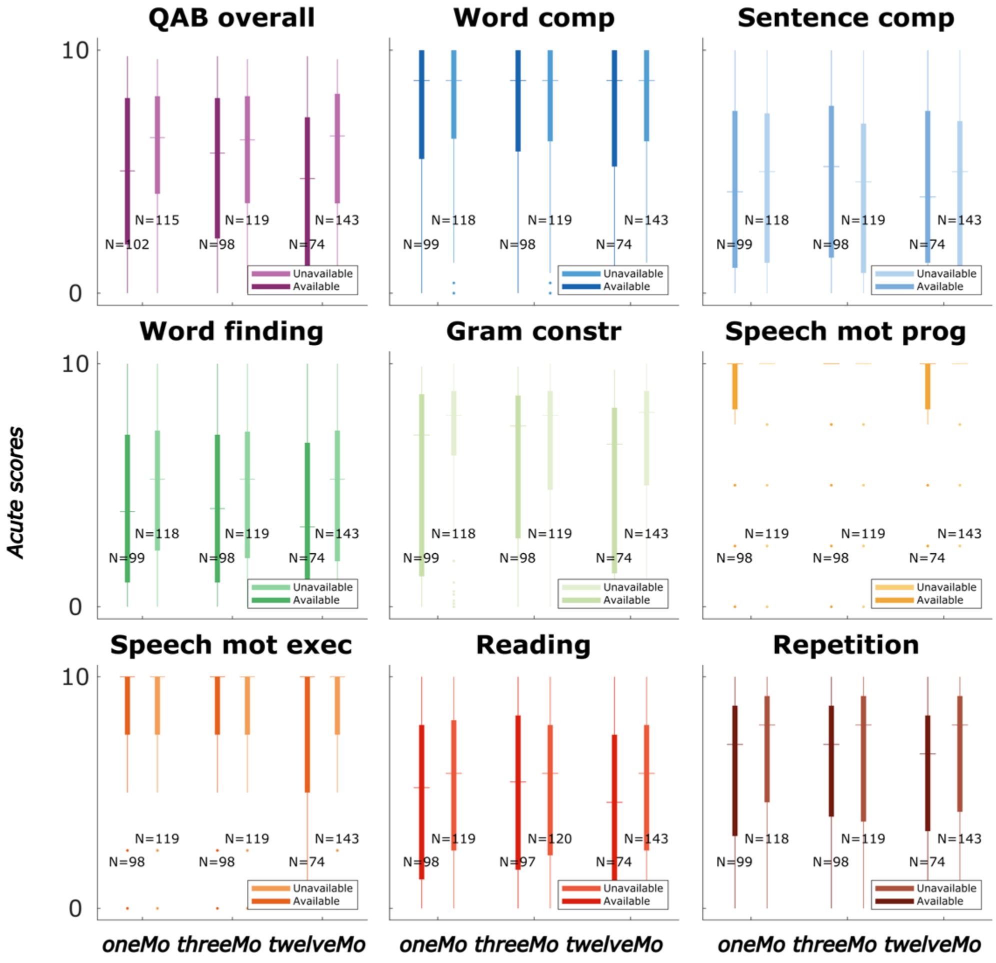

Supplementary Figure 2. Distributions of acute scores across timepoints. Box plots representing distributions of acute scores on each subscore for patients with and without data at each follow-up timepoint. Note minimal differences in these distributions in followed-up patients across timepoints, suggesting no longitudinal sampling bias towards patients who were less impaired acutely. QAB overall = Quick Aphasia Battery overall score; Word comp = single word comprehension; Sentence comp = sentence comprehension; Gram constr = grammatical construction; Speech mot prog = speech motor programming; Speech mot exec = speech motor execution.

LLV model performance					
	N	*r^2 dem*	*r^2 dem+les*	*r^2 full*	*RMSE full*
QAB, acute	217	-0.10	0.27	0.38	2.38
QAB, 1mo	102	-0.09	0.36	0.41	2.01
QAB, 3mo	98	-0.15	0.30	0.46	1.61
QAB, 12mo	74	-0.22	0.28	0.59	1.22
Word comp, acute	198	-0.16	0.05	0.18	2.80
Word comp, 1mo	99	-0.21	-0.10	0.04	2.54
Word comp, 3mo	98	-0.16	-0.01	0.04	1.75
Word comp, 12mo	74	-0.13	0.06	0.23	1.06
Sentence comp, acute	199	-0.03	0.15	0.15	3.11
Sentence comp, 1mo	99	-0.17	-0.04	0.21	3.02
Sentence comp, 3mo	98	-0.18	0.11	0.31	2.63

Sentence comp, 12mo	74	-0.25	-0.06	0.35	2.47
Word finding, acute	199	-0.11	0.18	0.25	2.67
Word finding, 1mo	99	-0.12	0.22	0.28	2.56
Word finding, 3mo	98	-0.15	0.28	0.51	1.99
Word finding, 12mo	74	-0.14	0.24	0.39	1.91
Gram constr, acute	199	-0.20	0.05	0.34	2.83
Gram constr, 1mo	99	-0.09	0.15	0.12	2.38
Gram constr, 3mo	98	-0.19	0.19	0.42	1.82
Gram constr, 12mo	74	-0.19	0.26	0.58	1.47
Speech mot prog, acute	184	-0.14	-0.14	0.28	2.15
Speech mot prog, 1mo	98	-0.18	-0.18	0.15	2.24
Speech mot prog, 3mo	98	-0.17	-0.13	0.43	1.85
Speech mot prog, 12mo	74	-0.20	-0.09	0.57	1.58
Speech mot exec, acute	187	-0.24	-0.24	0.30	2.16

Speech mot exec, 1mo	98	-0.14	-0.14	0.34	1.26
Speech mot exec, 3mo	98	-0.12	-0.12	0.18	1.07
Speech mot exec, 12mo	74	-0.09	-0.11	0.13	0.94
Reading, acute	194	-0.10	0.13	0.32	2.77
Reading, 1mo	98	-0.05	0.15	0.21	2.83
Reading, 3mo	97	-0.19	0.05	0.21	2.79
Reading, 12mo	74	-0.14	0.08	0.10	2.58
Repetition, acute	199	-0.14	0.11	0.36	2.68
Repetition, 1mo	99	-0.04	0.12	0.20	2.48
Repetition, 3mo	98	-0.15	0.24	0.26	2.11
Repetition, 12mo	74	-0.10	0.15	0.39	1.61

Supplementary Table 1. Sample size and metrics of prediction accuracy for the LLV (Lesion Load Vector) models across summary scores and time points. N=number of participants; dem=demographic variables; les=lesion size; full=all model variables including demographic, lesion size, and lesion load vectors; RMSE=root mean squared error; mo=months; QAB=Quick Aphasia Battery overall score; word comp=single word comprehension score; sentence comp=sentence comprehension score; gram constr=grammatical construction score; speech mot prog=speech motor programming score; speech mot exec=speech motor execution score.

**LLV+IP model performance**					
	N	*r^2 dem*	*r^2 dem+les*	*r^2 dem*	*RMSE full*
QAB, 1mo	102	0.60	0.73	0.64	1.58
QAB, 3mo	98	0.50	0.56	0.58	1.42
QAB, 12mo	74	0.24	0.36	0.60	1.21
Word comp, 1mo	99	-0.03	0.04	0.07	2.50
Word comp, 3mo	98	-0.04	0.06	0.09	1.70
Word comp, 12mo	74	-0.11	0.04	0.23	1.06
Sentence comp, 1mo	99	0.40	0.42	0.40	2.63
Sentence comp, 3mo	98	0.42	0.48	0.48	2.29
Sentence comp, 12mo	74	0.08	0.12	0.34	2.48
Word finding, 1mo	99	0.59	0.62	0.59	1.94
Word finding, 3mo	98	0.53	0.61	0.64	1.72

Word finding, 12mo	74	0.21	0.36	0.40	1.90
Gram constr, 1mo	99	0.45	0.49	0.46	1.87
Gram constr, 3mo	98	0.34	0.41	0.46	1.75
Gram constr, 12mo	74	0.15	0.33	0.59	1.45
Speech mot prog, 1mo	98	-0.17	-0.17	0.19	2.18
Speech mot prog, 3mo	98	-0.15	-0.10	0.42	1.87
Speech mot prog, 12mo	74	-0.10	0.02	0.57	1.57
Speech mot exec, 1mo	98	-0.14	-0.14	0.37	1.23
Speech mot exec, 3mo	98	-0.12	-0.12	0.15	1.09
Speech mot exec, 12mo	74	-0.09	-0.12	0.09	0.96
Reading, 1mo	98	0.27	0.37	0.28	2.69
Reading, 3mo	97	0.09	0.19	0.24	2.73
Reading, 12mo	74	-0.04	0.04	0.19	2.44
Repetition, 1mo	99	0.41	0.47	0.39	2.17

Repetition, 3mo	98	0.31	0.46	0.33	2.02
Repetition, 12mo	74	0.09	0.19	0.40	1.60

Supplementary Table 2. Sample size and metrics of prediction accuracy for the LLV+IP (Lesion Load Vector + Initial Presentation) models across summary scores and time points (note that the acute timepoint is excluded as scores from the acute timepoint were included as model predictors). N=number of participants; dem=demographic variables including acute QAB overall score; les=lesion size; full=all model variables including demographic, lesion size, and lesion load vectors; RMSE=root mean squared error; mo=months; QAB=Quick Aphasia Battery overall score; word comp=single word comprehension score; sentence comp=sentence comprehension score; gram constr=grammatical construction score; speech mot prog=speech motor programming score; speech mot exec=speech motor execution score.
